# Appraising the causal association between Crohn’s disease and breast cancer: a Mendelian randomization study

**DOI:** 10.3389/fonc.2023.1275913

**Published:** 2024-02-09

**Authors:** Chengdong Yu, Jiawei Xu, Siyi Xu, Yanxiao Huang, Lei Tang, Xiaoqiang Zeng, Tenghua Yu, Wen Chen, Zhengkui Sun

**Affiliations:** ^1^ Jiangxi Medical College, Nanchang University, Nanchang, China; ^2^ Department of Breast Surgery, Jiangxi Cancer Hospital, Nanchang, China

**Keywords:** Crohn’s disease, breast cancer, Mendelian randomization, causal association, risk

## Abstract

**Background:**

Previous research has indicated that there may be a link between Crohn’s disease (CD) and breast cancer (BC), but the causality remains unclear. This study aimed to investigate the causal association between CD and BC using Mendelian randomization (MR) analysis.

**Methods:**

The summary data for CD (5,956 cases/14,927 controls) was obtained from the International Inflammatory Bowel Disease Genetics Consortium (IIBDGC). And the summary data for BC (122,977 cases/105,974 controls) was extracted from the Breast Cancer Association Consortium (BCAC). Based on the estrogen receptor status, the cases were classified into two subtypes: estrogen receptor-positive (ER^+^) BC and estrogen receptor-negative (ER^−^) BC. We used the inverse variance weighted method as the primary approach for two-sample MR. MR-PRESSO method was used to rule out outliers. Heterogeneity and pleiotropy tests were carried out to improve the accuracy of results. Additionally, multivariable MR was conducted by adjusting for possible confounders to ensure the stability of the results.

**Results:**

The two-sample MR indicated that CD increased the risks of overall (OR: 1.020; 95% CI: 1.010-1.031; *p*=0.000106), ER^+^ (OR: 1.019; 95%CI: 1.006-1.034; *p*=0.006) and ER^−^ BC (OR: 1.019; 95%CI: 1.000-1.037; *p*=0.046) after removal of outliers by MR-PRESSO. This result was reliable in the sensitivity analysis, including Cochran’s Q and MR-Egger regression. In multivariate MR analyses, after adjusting for smoking and drinking separately or concurrently, the positive association between CD and the risks of overall and ER^+^ BC remained, but it disappeared in ER^−^ BC. Furthermore, reverse MR analysis suggested that BC did not have a significant impact on CD risk.

**Conclusion:**

Our findings provide evidence for a possible positive association between CD and the risk of BC. However, further studies are needed to fully understand the underlying mechanisms and establish a stronger causal relationship.

## 1 Introduction

Crohn’s disease (CD) is a chronic and progressive inflammatory disease characterized by alternating periods of remission and relapse (1, 2). CD primarily affects the gastrointestinal tract with extraintestinal manifestations and related immune dysregulation (3). Patients with CD are more susceptible to cancer, depression, and infection (4).

Breast cancer (BC) is the most prevalent malignancy among women globally, with 684,996 deaths reported in 2020, representing a substantial threat to their health (5–7). Patients with CD have an increased risk of digestive tract, skin, bladder, and lung cancers (8, 9), but the association between CD and BC remains unclear. Chronic inflammation characterized by sustained immune activation is associated with promoting the occurrence, growth, and progression of BC (10–12). Several researchers have investigated the association between CD and BC. Riegler et al. found first-degree relatives of patients with CD have a higher risk of developing BC (13). Further, a study by Pellino et al. showed that CD was an independent risk factor for BC (OR: 2.76; 95% CI: 1.2-6.2; *p*=0.017) *(* 14). In contrast, Gong et al. reported no significant association between CD and BC risk (15). Hence, there is controversy regarding the relationship between CD and BC risk. In addition, immunosuppressive medications are the cornerstone of long-term maintenance treatment for CD (16). Due to the decreased immune surveillance, immunosuppression may potentially increase the risk of cancer (17). A retrospective study attributed the development of BC in CD patients to immunosuppressive therapy (18). Thus, the association between CD itself and BC needs to be further investigated. Moreover, assessing the true causal association between CD and BC is challenging due to the interference of common residual confounders and reverse causality in traditional observational studies.

To overcome these challenges and gain a more nuanced understanding of the causality between CD and BC, we turned to Mendelian randomization (MR). MR is a robust statistical method that harnesses genetic variants as instrumental variables (IVs) to explore causal connections between exposure and outcome (19, 20). By capitalizing on the natural random assortment of genetic variants during conception, MR effectively mimics the randomized controlled trial (RCT) setting, thereby mitigating issues like confounding and reverse causation that often plague observational studies (21–23).

## 2 Materials and methods

### 2.1 Study design

**Figure 1:**
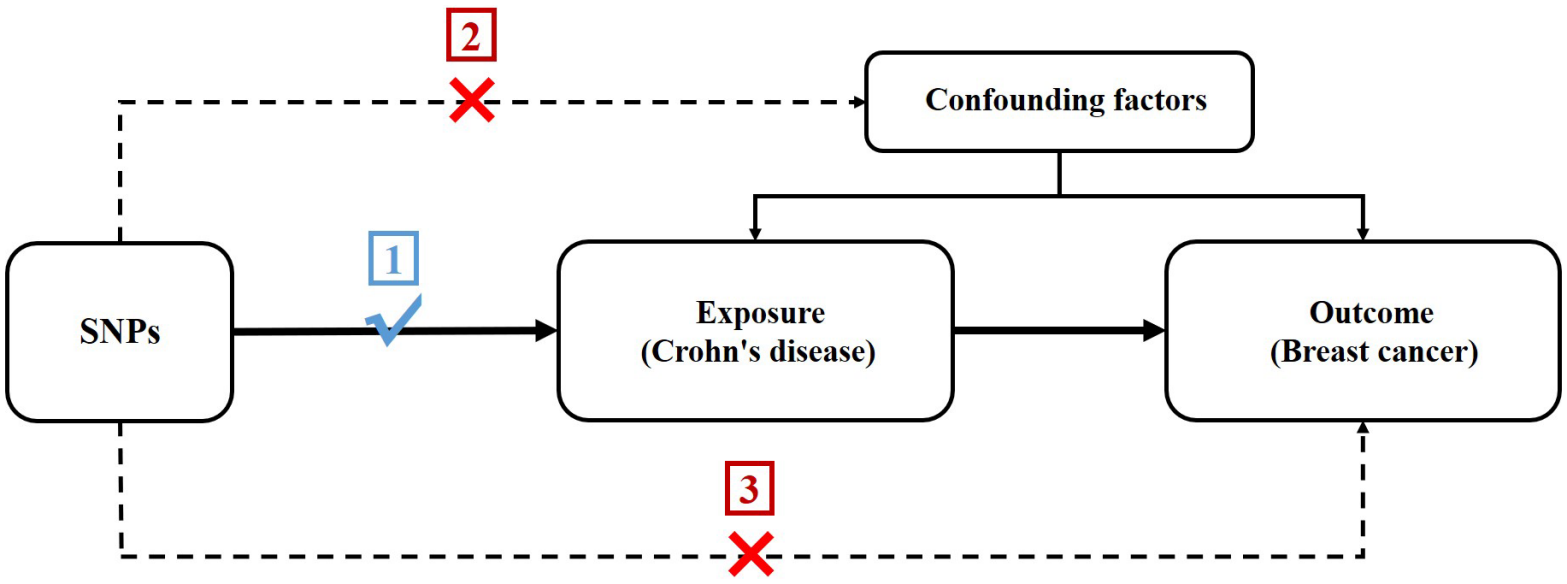
Schematic for the MR study design.

In order to assess the potential causal association between CD and BC, we conducted a two-sample MR study. The single nucleotide polymorphisms (SNPs) selected as IVs were required to adhere to three following key premises (24): (1) SNPs must be intensely linked to CD; (2) SNPs must not be linked to confounding factors; and (3) SNPs should not be directly linked to BC (**Figure 1**).

### 2.2 Data source

The summary data for CD (5,956 cases/14,927 controls) was obtained from the International Inflammatory Bowel Disease Genetics Consortium (IIBDGC) (25). And the summary data for BC (122,977 cases/105,974 controls) was extracted from the Breast Cancer Association Consortium (BCAC). Based on the estrogen receptor status, the cases were classified into two subtypes: estrogen receptor-positive (ER^+^) BC and estrogen receptor-negative (ER^−^) BC (26). **Table 1** presents details of the exposure and outcomes.

**Table 1:** Detailed information on the exposure and outcomes.

Exposure/Outcome	ncase	ncontrol	Sample size	Consortium	Ancestry
Crohn’s disease	5,956	14,927	20,883	IIBDGC	European
Overall Breast cancer	122,977	105,974	228,951	BCAC	European
ER^+^ Breast cancer	69,501	105,974	175,475	BCAC	European
ER^−^ Breast cancer	21,468	105,974	127,442	BCAC	European

BCAC, Breast Cancer Association Consortium; IIBDGC, International Inflammatory Bowel Disease Genetics Consortium.

### 2.3 SNP selection

**Figure 2:**
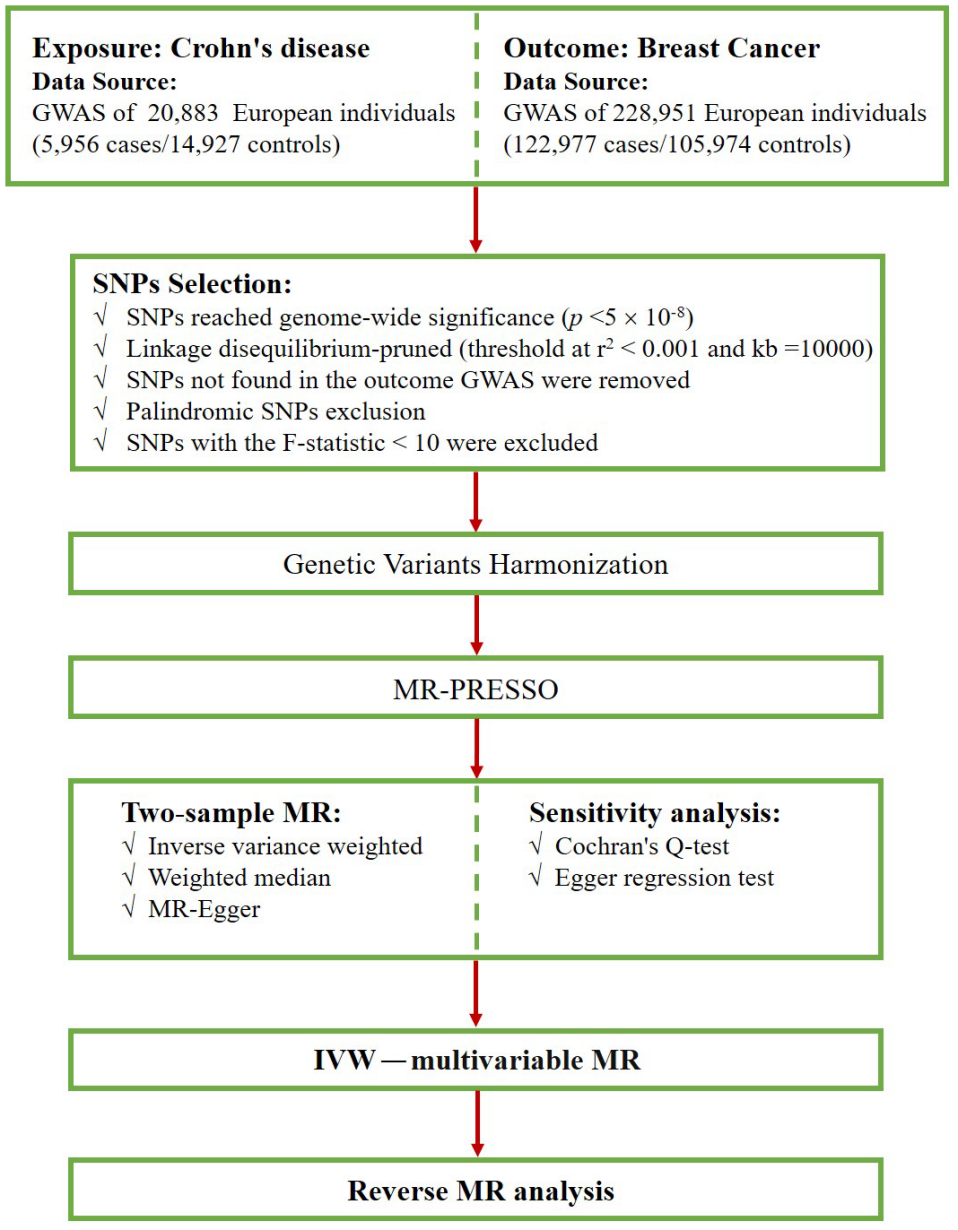
Flowchart of our MR study.

First, we screened for SNPs that were strongly associated with exposure at a genome-wide significance level (*p *< 5×10^–8^). Second, we implemented a criterion (r^2^ < 0.001, kb=10000) to select SNPs that were independent of linkage disequilibrium (LD) (27). Third, we excluded SNPs that were not found in the BC dataset and palindromic SNPs that may cause bias. Next, we harmonized the exposure and outcome data, ensuring that the effect of the SNP on the exposure corresponded to the same allele as the effect on the outcome. Subsequently, we evaluated the possibility of weak instrumental bias by calculating F-statistics and excluded SNPs with F-statistics less than 10 (28, 29). The F statistic was calculated as F = beta^2^/se^2^ (30, 31). Finally, the MR-PRESSO method was conducted to detect outlier SNPs (32), and after excluding these outlier SNPs, the remaining SNPs were used for subsequent MR analysis. **Figure 2** shows the selection flowchart.

### 2.4 Two-sample MR analysis

Three commonly used MR methods were applied to estimate causal effects: inverse variance weighted (IVW) (24), weighted median (33), and MR-Egger (34). The IVW method is considered to be the most effective method for assessing causality (35); therefore, the results were mainly based on the IVW method. We used odds ratios (ORs) to express the effects of CD on BC risk. If the result of the IVW method is significant (*p* < 0.05), even if no significant result is obtained by the other methods, it can be considered as a positive result as long as the ORs of the other methods are in the same direction and there is no heterogeneity or pleiotropy (36).

### 2.5 Sensitivity analysis

Cochran’s Q test was employed to assess heterogeneity, with *p* > 0.05 representing the absence of heterogeneity (37). The MR-Egger regression test was applied to detect horizontal pleiotropy, with a zero intercept signifying the absence of pleiotropy (*p* > 0.05) (38).

### 2.6 Multivariable MR analysis

Based on the search results on the PhenoScanner website and possible confounders between CD and BC, we performed multivariable MR (MVMR) analyses with the addition of smoking and drinking separately or together to adjust for causal impacts between exposure and outcome (39).

### 2.7 Reverse MR analysis

To explore whether BC has any causal effect on CD, we also conducted a reverse MR analysis (i.e., BC as the exposure and CD as the outcome) using SNPs related to BC as IVs.

### 2.8 Statistical analyses

All analyses were performed in R software (version 4.2.3) using the “TwoSampleMR” (version 0.5.6), “MRPRESSO” (version 1.0), and “MendelianRandomization” (version 0.7.0) packages (40).

## 3 Results

### 3.1 SNP selection

Initially, we extracted 53 genome-wide significant (*p*<5×10^-8^) SNPs associated with CD. No SNPs were ruled out due to LD. Next, during the extraction of information on IVs and outcome, we excluded rs11564236 due to the lack of corresponding outcome data. Additionally, we excluded one palindromic SNP (rs12692254) while harmonizing the exposure and outcome data. Furthermore, we removed rs7543234 from the analysis of overall BC due to its association with the outcome. Finally, potentially outlier SNPs were excluded using MR-PRESSO. Specifically, rs12194825, rs1873625, rs2188962, and rs3091315 were excluded from the analysis of overall BC; rs12194825, rs1873625, rs2188962, and rs7543234 were excluded from the analysis of ER^+^ BC, and rs1873625 and rs3091315 were removed from the analysis of ER^−^ BC. The F-statistics of all SNPs were greater than 10. After removing these SNPs, 46 SNPs, 47 SNPs, and 49 SNPs were included in the analysis of overall, ER^+^, and ER^−^ BC, respectively (**Supplementary Sheet**).

### 3.2 Analyses using the Two-sample MR

**Figure 3:**
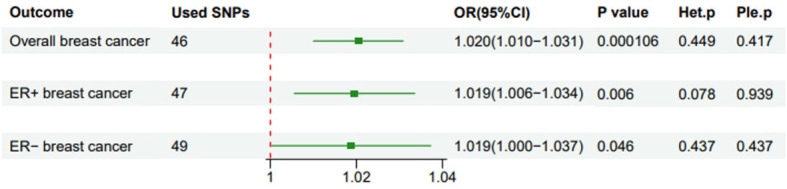
Forest plot of the impact of Crohn’s disease on breast cancer risk using IVW method (after removing outliers). Het.p refers to the p-value for heterogeneity; Ple.p refers to the p-value for pleiotropy; OR, odds ratio; CI, confidence interval.

**Figure 4:**
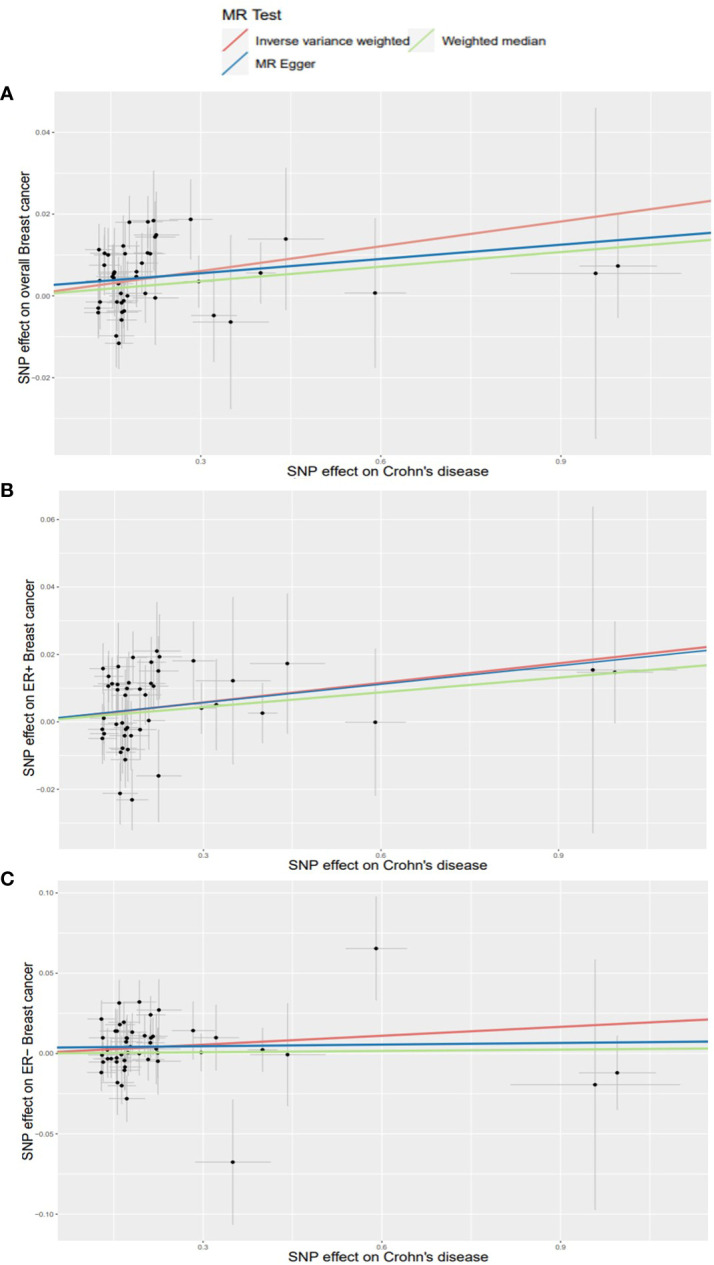
Scatterplots for effects of Crohn’s disease on breast cancer risk (after removing outliers). **(A)** overall BC; **(B)** ER^+^ BC; **(C)** ER^−^ BC.

**Figure 5:**
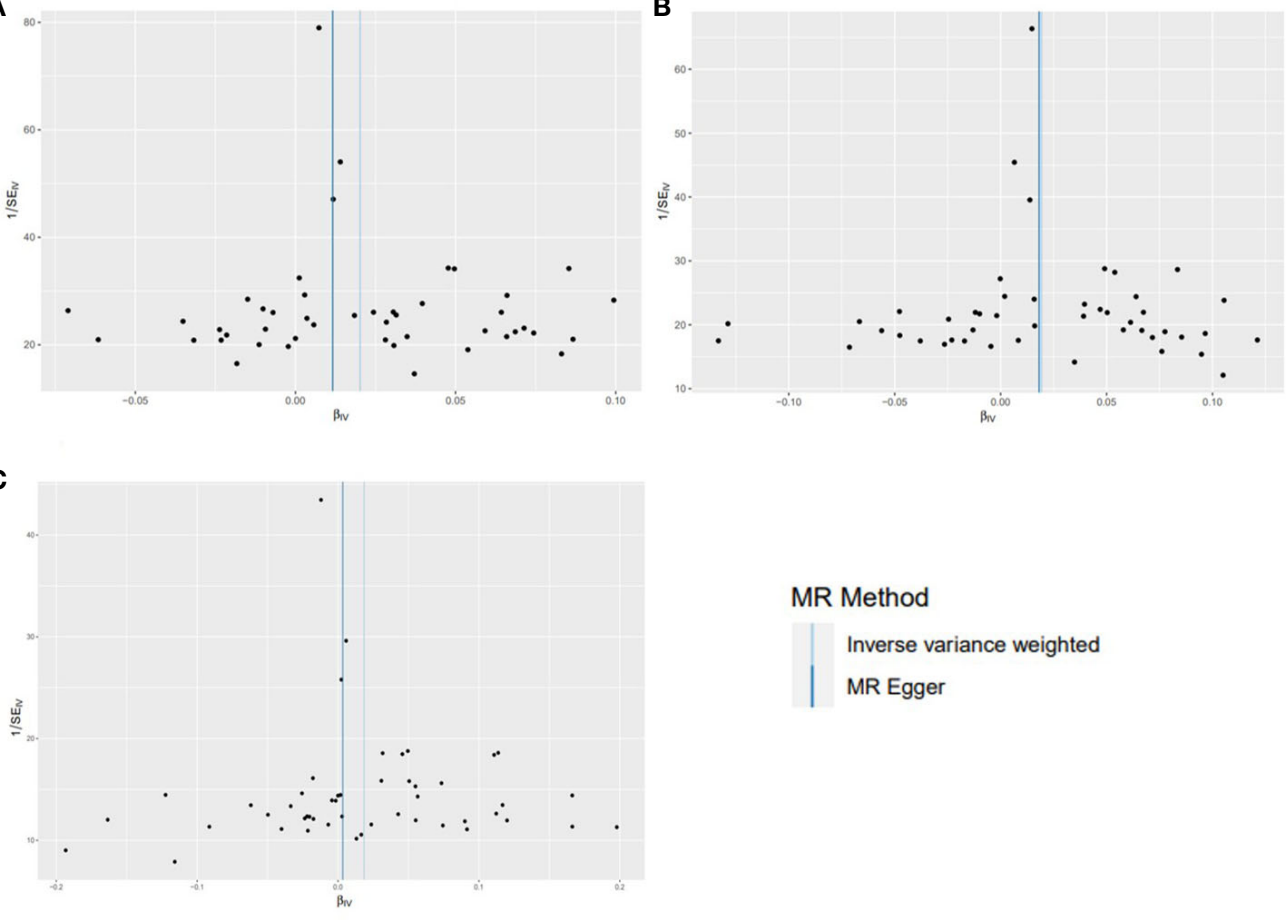
Funnel plots for effects of Crohn’s disease on breast cancer risk (after removing outliers). **(A)** overall BC; **(B)** ER^+^ BC; **(C)** ER^−^ BC.

Using existing SNPs as IV, the results of the IVW method showed that CD was positively associated with the risks of overall (OR: 1.020; 95% CI: 1.010-1.031; *p*=0.000106), ER^+^ (OR: 1.019; 95% CI: 1.006-1.034; *p*=0.006), and ER^−^ (OR: 1.019; 95% CI: 1.000-1.037; *p*=0.046) BC (**Figure 3**). The scatterplot depicts the causal estimates obtained from every SNP (**Figure 4**). Although the weighted median and MR-Egger methods did not obtain significant results (*p* > 0.05), the direction of the ORs was consistent with the IVW method (OR > 1). Furthermore, Cochran’s Q and MR-Egger regression analyses demonstrated that there was no heterogeneity or horizontal pleiotropy affecting the stability of the results. The same result was also suggested by the symmetry of the funnel plots (**Figure 5**). Therefore, based on the significant IVW results (*p* < 0.05), we can conclude that there is a causal effect of CD on BC. The details of the results are presented in **Table 2**.

**Table 2:** Assessing the effects of Crohn’s disease on breast cancer risk (after removing outliers).

Outcome	MR-PRESSO Outliers	Used SNPs	method	OR (95%CI)	*P*	Het.p	Ple.p
Overall breast cancer	rs12194825, rs1873625, rs2188962, rs3091315	46	IVW	1.020(1.010-1.031)	0.000106	0.449	0.417
			weighted median	1.012(0.996-1.029)	0.152		
			MR-Egger	1.012(0.989-1.035)	0.326		
ER^+^ breast cancer	rs12194825, rs1873625, rs2188962, rs7543234	47	IVW	1.019(1.006-1.034)	0.006	0.078	0.939
			weighted median	1.015(0.995-1.035)	0.144		
			MR-Egger	1.018(0.987-1.051)	0.261		
ER^−^ breast cancer	rs1873625, rs3091315	49	IVW	1.019(1.000-1.037)	0.046	0.437	0.437
			weighted median	1.003(0.976-1.031)	0.846		
	,		MR-Egger	1.003(0.962-1.046)	0.874		

Het.p refers to the p-value for heterogeneity; Ple.p refers to the p-value for pleiotropy; OR, odds ratio; CI, confidence interval.

### 3.3 Analyses using the MVMR

After adjusting for current tobacco smoking and alcoholic drinks per week separately or together, MVMR analysis revealed that the positive association between CD and the risks of overall and ER^+^ BC remained, but it disappeared in ER^−^ BC. In addition, no potential horizontal pleiotropy was discovered for the MR-Egger intercept (**Table 3**). Results of MVMR suggested that the observed effects of CD on overall and ER^+^ BC were stable and not influenced by potential confounders.

**Table 3:** Assessing the effects of Crohn’s disease on breast cancer using IVW multivariable MR.

Outcome	Adjustment	OR (95%CI)	*P*	Egger-Intercept	Int.p
Overall breast cancer	Current tobacco smoking	1.018(1.003-1.033)	0.019	<0.001	0.976
	Alcoholic drinks per week	1.019(1.001-1.037)	0.037	-0.001	0.724
	Adjust together	1.017(1.001-1.034)	0.037	-0.001	0.586
ER^+^ breast cancer	Current tobacco smoking	1.023(1.005-1.040)	0.011	0.001	0.560
	Alcoholic drinks per week	1.021(1.002-1.041)	0.034	<0.001	0.960
	Adjust together	1.021(1.003-1.039)	0.025	<0.001	0.982
ER^−^ breast cancer	Current tobacco smoking	1.002(0.980-1.024)	0.864	-0.002	0.555
	Alcoholic drinks per week	1.003(0.978-1.028)	0.833	-0.005	0.153
	Adjust together	0.998(0.975-1.022)	0.869	-0.003	0.164

Int.p refers to the p-value derived from the Egger-intercept.

### 3.4 Reverse MR analysis

In the reverse study (BC on the risk of CD), no genetic effects of overall BC (OR: 1.082; 95% CI: 0.989-1.183; *p*=0.085), ER^+^ BC (OR: 1.039; 95% CI: 0.950-1.136; *p*=0.405), and ER^−^ BC (OR: 1.033; 95% CI: 0.924-1.156; *p*=0.567) on the risk of CD were detected (**Table 4**). In all of the analyses, MR-Egger regression did not show IVs had horizontal pleiotropy. Therefore, genetically predicted BC exerts no impact on the risk of CD.

**Table 4:** Assessing the effects of breast cancer on Crohn’s disease using IVW method.

Expose	Outcome	Used SNPs	OR (95%CI)	*P*	Egger-Intercept	Int.p
Overall breast cancer	Crohn’s disease	132	1.082(0.989-1.183)	0.085	0.002	0.790
ER^+^ breast cancer	Crohn’s disease	97	1.039(0.950-1.136)	0.405	-0.006	0.431
ER^−^ breast cancer	Crohn’s disease	34	1.033(0.924-1.156)	0.567	-0.018	0.293

Int.p refers to the p-value derived from the Egger-intercept.

## 4 Discussion

In this study, we carried out two-sample MR analyses to appraise the causal relationship of CD with overall, ER^+^, and ER^−^ BC for the first time. The results showed that CD increased the risks of overall, ER^+^, and ER^−^ BC. We further assessed the robustness of the results by MVMR analysis. However, in MVMR analysis, CD only increased the risks of overall and ER^+^ BC, but not ER^−^ BC. This suggested a potential impact of smoking and alcohol drinking on the correlation between CD and ER^−^ BC. Additionally, reverse MR analysis revealed that BC did not have a significant impact on CD risk.

However, a recent MR study found no association between CD and BC risk (41). We analyzed possible reasons for the discrepancy. Their study included only 732 cases of CD, whereas our study included 5,956 cases. They used a significance threshold of *p*<5×10^-6^ for SNP selection, but re-running MR on their data at *p*<5×10^-8^ revealed a positive association of CD on BC risk (*p*=0.016). Furthermore, we conducted subtype analyses based on estrogen receptor status and performed MVMR to adjust for possible confounding factors.

This MR study provides some insights into the association between CD and BC. Some studies have also revealed an elevated risk of BC in patients with CD (13, 14). The result of a 20-year follow-up study indicated that CD patients have a higher risk of developing BC (42). In addition, a study from Denmark showed BC patients with CD have a more advanced stage and a worse chemotherapy prognosis than patients without CD (43).

Several possible factors may account for the association between CD and BC. Existing studies indicated that CD and BC may share common molecular mechanisms. Recent evidence suggested that there are 53 overlapping differentially expressed genes between the CD and BC, with enrichment analyses showing that both diseases are related to NF-κB signaling pathways and interleukin-17 (IL-17) (44). It has been shown that inflammation is involved in the process of development and progression of malignant tumors (45). T helper 17 (Th17) cells are important inflammatory mediators in CD, and when Th17 cells reach breast tumor tissues, they upregulate a variety of cytokines including IL-17 and tumor necrosis factor-α (TNF-α) (46). IL-17 can upregulate the expression of chemokine CXCL1 in BC cells. This chemokine increases the activation of the AKT/NF-κB signaling pathway to promote BC growth and metastasis (47). Furthermore, previous studies have indicated that TNF-α is involved in epithelial-mesenchymal transition (EMT), thereby promoting tumor metastasis (48). A study conducted on patients with inflammatory BC demonstrated a direct association between TNF-α and the presence of tumor cells expressing EMT markers (49). In addition, there is another potential point of association between CD and BC that lies in the involvement of estrogen and the G protein-coupled estrogen receptor (GPER) (50, 51). GPER has been shown to regulate intestinal function, inflammation, and immune responses, and promote the occurrence and progression of BC (52, 53).

There is growing interest in the role of the microbiome in health and disease. Studies in human subjects have revealed distinct differences in the gut microbiome between patients with CD and healthy control subjects (54). Notably, the gut microbiome also affects the risk of developing BC (55). Dysbiosis of the intestinal flora has been found to have a direct effect on the dissemination of breast tumors (56, 57). The gut microbiome may also be involved in the correlation between CD and the risk of BC, and more relevant research is needed to confirm this in the future.

**Figure 6:**
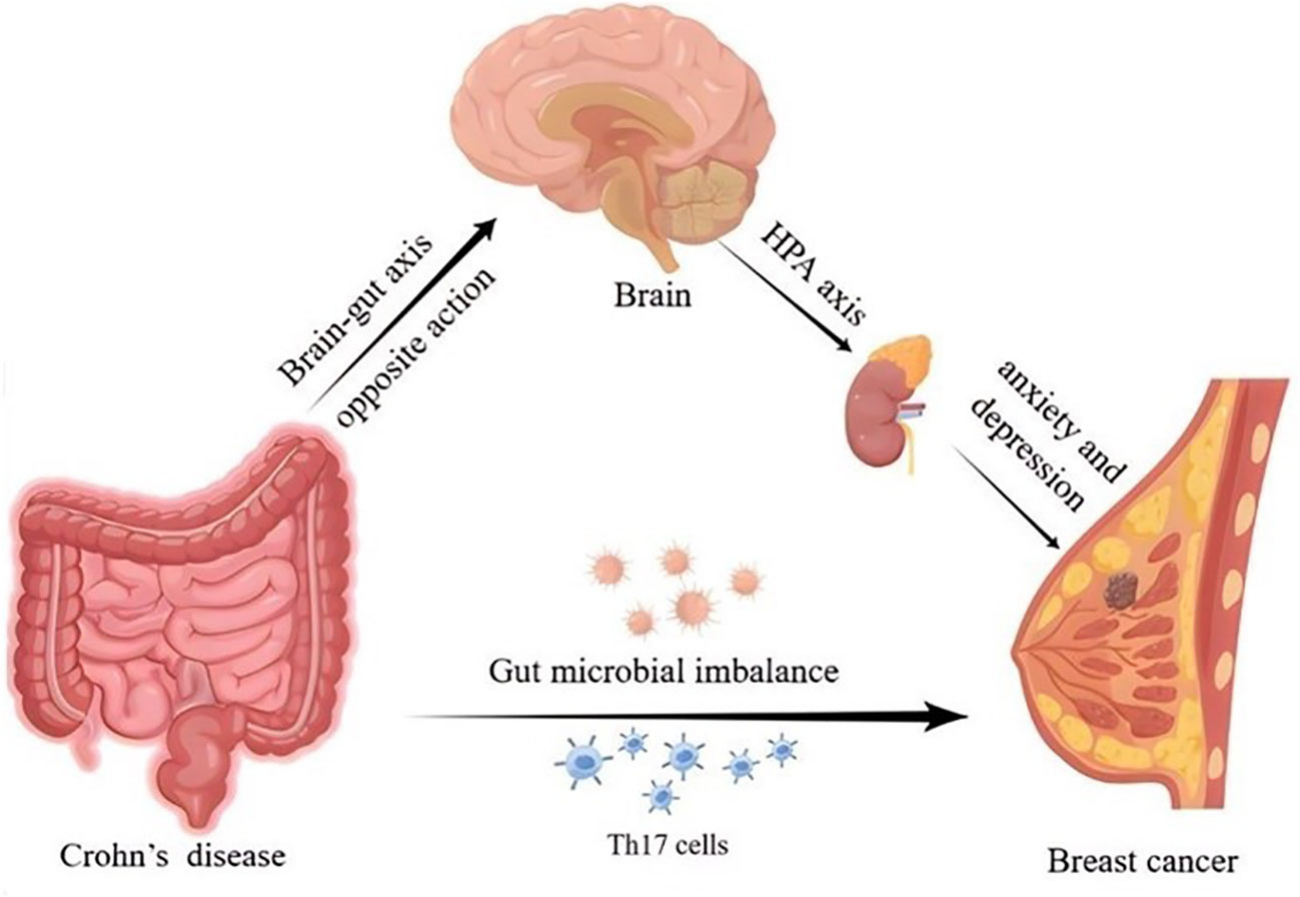
Diagram of possible mechanisms for the effect of Crohn’s disease on breast cancer risk.

Research has demonstrated that chronic psychological stress can inhibit the anti-tumor effects of the immune system in CD (58). Intestinal inflammation in CD can activate the hypothalamic–pituitary–adrenal (HPA) axis through the opposite action of the brain-gut axis, thereby inducing anxiety and depression (59, 60). Several studies have shown that patients with BC also experience varying degrees of anxiety and depression (61). Hence, the mechanisms behind the effects of mental and emotional factors on CD and BC need to be further explored. The possible mechanisms for the effect of CD on BC risk are depicted in **Figure 6**.

The strength of our study is that it explored the causality between CD and BC risk by MR analysis. Compared to previous observational studies that found CD to be associated with BC, MR studies are less susceptible to confounders and reverse causation. Besides, our study utilized a large sample size and SNPs from GWAS, providing sufficient statistical validity to estimate causality. Furthermore, sensitivity analyses enhanced the credibility of our results.

Nevertheless, this study has several limitations. First, the GWAS data for this study included only European populations, which limits the application of our findings to other populations. Hence, future studies are required to verify the applicability of our results to different populations. Second, we cannot stratify the analysis by sex due to the lack of sex-specific GWAS data. Third, the OR of CD on BC risk is relatively small, indicating that the enhanced risk is just modest. Therefore, we don’t recommend that patients with CD be screened for BC more frequently or earlier than the routine screening. Finally, MR also has its limitations. (1) SNPs are generally considered to have lifetime effects, but in specific situations, the effects of SNPs may vary due to an individual’s physiological status, environmental factors, or interactions with other genetic variations. If the genetic variants used in MR analysis change over time, it could potentially affect the validity of the causal estimates. (2) Additional adjustments for smoking and alcohol consumption may lead to collider bias. (3) The MR study can only analyze the causality and cannot explain the mechanism of CD on BC risk. Further research is necessary to investigate the mechanisms behind the link between CD and the risk of BC.

## 5 Conclusion

Our findings provide evidence for a potential positive association between CD and the risk of BC. However, further studies are needed to fully understand the underlying mechanisms and establish a stronger causal relationship.

## Data availability statement

In this study, all GWAS data were extracted from the IEU Open GWAS project (https://gwas.mrcieu.ac.uk/).

## Author contributions

CY: Conceptualization, Investigation, Methodology, Writing – original draft. JX: Data curation, Writing – original draft. SX: Data curation, Writing – review & editing. YH: Writing – review & editing. LT: Writing – review & editing. XZ: Writing – review & editing. TY: Formal Analysis, Writing – original draft. WC: Visualization, Writing – review & editing. ZS: Funding acquisition, Supervision, Validation, Writing – review & editing.
